# LncRNA-miRNA interaction prediction through sequence-derived linear neighborhood propagation method with information combination

**DOI:** 10.1186/s12864-019-6284-y

**Published:** 2019-12-20

**Authors:** Wen Zhang, Guifeng Tang, Shuang Zhou, Yanqing Niu

**Affiliations:** 1 College of informatics, Huazhong Agricultural University, Wuhan, 430070 China; 2 School of Computer Science, Wuhan University, Wuhan, 430072 China; 3 Department of Computer Science and Engineering, The Chinese University of Hong Kong, Hong Kong, China; 4 School of Mathematics and Statistics, South-Central University for Nationalities, Wuhan, 430074 China

**Keywords:** lncRNA-miRNA interactions, Integrated similarity, Label propagation

## Abstract

**Background:**

Researchers discover lncRNAs can act as decoys or sponges to regulate the behavior of miRNAs. Identification of lncRNA-miRNA interactions helps to understand the functions of lncRNAs, especially their roles in complicated diseases. Computational methods can save time and reduce cost in identifying lncRNA-miRNA interactions, but there have been only a few computational methods.

**Results:**

In this paper, we propose a sequence-derived linear neighborhood propagation method (SLNPM) to predict lncRNA-miRNA interactions. First, we calculate the integrated lncRNA-lncRNA similarity and the integrated miRNA-miRNA similarity by combining known lncRNA-miRNA interactions, lncRNA sequences and miRNA sequences. We consider two similarity calculation strategies respectively, namely similarity-based information combination (SC) and interaction profile-based information combination (PC). Second, the integrated lncRNA similarity-based graph and the integrated miRNA similarity-based graph are respectively constructed, and the label propagation processes are implemented on two graphs to score lncRNA-miRNA pairs. Finally, the weighted averages of their outputs are adopted as final predictions. Therefore, we construct two editions of SLNPM: sequence-derived linear neighborhood propagation method based on similarity information combination (SLNPM-SC) and sequence-derived linear neighborhood propagation method based on interaction profile information combination (SLNPM-PC). The experimental results show that SLNPM-SC and SLNPM-PC predict lncRNA-miRNA interactions with higher accuracy compared with other state-of-the-art methods. The case studies demonstrate that SLNPM-SC and SLNPM-PC help to find novel lncRNA-miRNA interactions for given lncRNAs or miRNAs.

**Conclusion:**

The study reveals that known interactions bring the most important information for lncRNA-miRNA interaction prediction, and sequences of lncRNAs (miRNAs) also provide useful information. In conclusion, SLNPM-SC and SLNPM-PC are promising for lncRNA-miRNA interaction prediction.

## Background

Non-coding RNAs (ncRNAs) are a class of RNAs that are not translated into functional proteins [1]. NcRNAs can be classified into many types, e.g. long non-coding RNA, circular RNA, snRNA, etc. Long non-coding RNAs (lncRNAs) are a kind of ncRNAs whose lengths are more than 200 nucleotides [2]. Studies [3, 4] show that a great number of lncRNAs are involved in many biological processes, such as cell proliferation, chromatin remodeling, gene imprinting and immune response. More importantly, some researchers discovered that lncRNAs are associated with severe diseases such as prostate cancer and gastric cancer [5–10].

LncRNAs play functional roles by interacting with other biological molecules (DNAs, RNAs and proteins), and the studies on lncRNA-biomolecule interactions help to characterize the functions of lncRNAs. For example, lncRNA loc285194 can interact with p53 gene and act as a tumor suppressor [11]; lncRNA PVT1 interacts with FOXM1 protein and promotes gastric cancer progression [12]. For a long time, researchers have been paying attention to lncRNA-DNA interactions [13, 14] or lncRNA-protein interactions [15, 16]. Recently, some researchers discover [17] that lncRNAs can act as decoys or sponges to regulate the behavior of miRNAs. For example, the lncRNA H19 is found to modulate let-7 family of miRNAs [18]. Therefore, exploring lncRNA-miRNA interactions contributes to understanding the complicated functions of lncRNAs.

Previous studies conduct wet experiments to identify lncRNA-miRNA interactions. For example, Amanda et al. [18] carry out in vivo crosslinking combined with affinity purification experiments to explore the interaction between lncRNA H19 and miRNA let-7. Based on the crosslinking and real-time PCR (RT-qPCR) experiment, their results demonstrated that lncRNA H19 can physically interact with let-7 in vivo. Zhang et al. [19] once studied the miRNA miR-7’s function in breast cancer stem cell (BCSCs) and its associated lncRNA. By implementing ChIP-PCR and Double-Luciferase Reporter assay, they find that the downregulation of miR-7 in BCSCs might be indirectly attributed to lncRNA HOTAIR. The wet methods are time-consuming and labor-intensive; thus, it is important to perform in silico prediction to refine the candidate list for further validation experiments.

Recently, researchers introduce machine learning techniques into the lncRNA-biomolecule interaction prediction, especially the lncRNA-protein interaction [20–25]. However, only a few lncRNA-miRNA interaction prediction methods have been proposed. Huang et al. [26] propose a method named EPLMI, which relies on the assumption that lncRNAs having similar expression profiles are prone to associate with a cluster of miRNAs that have similar expression profiles. Huang et al. [27] develop a novel group preference Bayesian collaborative filtering model called GBCF, which picks up a top-k probability ranking list for an individual miRNA or lncRNA based on known miRNA-lncRNA interaction network. Hu et al. [28] predict lncRNA-miRNA interactions by integrating the expression similarity network and the sequence similarity network, and develop a method named INLMI. Nevertheless, these methods have several limitations, which inspire us to develop better models. Firstly, existing methods rely on several features of lncRNAs and miRNAs, such as sequences, expression profiles and target genes, but expression profiles and target genes are not available for all lncRNAs (or miRNAs). Secondly, many lncRNAs and miRNAs do not have any known interaction, but a desirable model should be capable of predicting their interactions.

In this paper, we propose a sequence-derived linear neighborhood propagation method (SLNPM) to predict lncRNA-miRNA interactions. First, we calculate integrated lncRNA-lncRNA similarity and integrated miRNA-miRNA similarity by combining known lncRNA-miRNA interactions, lncRNA sequences and miRNA sequences. As the extension of our previous work [29], we consider two integrated similarity calculation strategies, namely similarity-based information combination (SC) and interaction profile-based information combination (PC). Second, the integrated lncRNA similarity-based graph and the integrated miRNA similarity-based graph are respectively constructed, and the label propagation processes are respectively implemented on two graphs to score lncRNA-miRNA pairs. Finally, the averages of their outputs are adopted as final predictions. In this way, we construct two editions of SLNPM based on similarity information combination (SLNPM-SC) and based on interaction profile information combination (SLNPM-PC). The experimental results show that SLNPM-SC and SLNPM-PC predict lncRNA-miRNA interactions with higher accuracy compared with other state-of-the-art methods. We also analyze the prediction capability of SLNPM-SC and SLNPM-PC for lncRNAs (or miRNAs) which do not have any known interaction, and the case studies demonstrate that SLNPM-SC and SLNPM-PC help to find novel interactions which do not exist in our dataset.

This paper makes the following contributions: (1) the proposed SLNPM models make use of diverse information to achieve high-accuracy performances; (2) the proposed SLNPM models can deal with the lncRNAs (or miRNAs) that do not have any known interaction.

## Datasets and methods

### Datasets

There are several datasets about lncRNAs, miRNAs and lncRNA-miRNA interactions, such as lncRNASNP [17], NONCODE [30], miRBase [31] and miRmine [32]. LncRNASNP [17] contains experimentally validated lncRNA-related SNPs and lncRNA-miRNA interactions, which can facilitate to study lncRNAs’ functions. NONCODE [30] is an integrated knowledge database of non-coding RNAs (ncRNAs). The ncRNA sequences and related information (e.g. function, cellular role, cellular location, chromosomal information, etc.) in NONCODE have been confirmed manually by consulting relevant literature. MiRBase [31] is a comprehensive database about miRNAs, containing published miRNA sequences and annotation. The database miRmine [32] provides high-quality human miRNA-Seq and miRNA expression profiles.

To compile our datasets, we first download data from lncRNASNP, and obtain 8091 experimentally verified lncRNA-miRNA interactions. After removing duplicated associations, there remain 5118 interactions between 780 lncRNAs and 275 miRNAs. Then, we collect lncRNA’s sequences from NONCODE and collect miRNAs’ sequences from miRbase. Thus, sequences are available for 642 lncRNAs and 275 miRNAs. Next, we obtain expression profiles of lncRNAs in 24 human tissues from NONCODE, and obtain expression profiles of miRNAs in 16 types of human tissues and 24 types of cell types from miRmine. The expression profiles are available for 417 lncRNAs and 265 miRNAs. Therefore, we compile a dataset named SLNPM-S by removing lncRNAs and miRNAs whose sequences or expression profiles are unavailable. Similarly, we compile a dataset named SLNPM-L by removing lncRNAs and miRNAs whose sequences are unavailable. SLNPM-S serves as the main dataset for model training and performance evaluation, and SLNPM-L is used for the case study. Table 1 summarizes the details of two datasets.

**Table 1:** Summary of SLNPM-S and SLNPM-L datasets

Dataset	LncRNAs	MiRNAs	Interactions	Features
SLNPM-S	417	265	2272	Sequences, Expression Profiles
SLNPM-L	642	275	3784	Sequences

### Linear neighborhood similarity measure

In previous work [33, 34], we proposed a novel similarity measure named linear neighborhood similarity (LNS), and successfully solved several problems in bioinformatics [24, 35–37]. In this paper, we adopt the linear neighborhood similarity measure (LNS) to calculate lncRNA-lncRNA similarity and miRNA-miRNA similarity. Here we first introduce the detailed process of LNS.

Given *n*-dimensional feature vectors *x*_1_, *x*_2_, ⋯, *x*_*m*_, these feature vectors are considered as the data points in the feature space. We concentrate the vectors row by row to obtain the *n* × *m* matrix *X*, where *x*_*i*_ is the *i* th row of the matrix *X*. It is assumed that each data point can be reconstructed by the linear weighted sum of neighboring data points. Generally, nearest neighbors based on the Euclidean distance are selected for each data point *x*_*i*_, and the ratio of the neighbors (selected nearest neighbors vs all neighboring data points) is called neighborhood ratio, denoted by *K*. *N*(*x*_*i*_) is the set of selected nearest neighbors of *x*_*i*_. By minimizing the reconstructive errors for all data points, we present the following optimization problem:

1 $$ \underset{W}{\mathit{\min}}\frac{1}{2}{\left\Vert X-\left(C\odot W\right)X\right\Vert}_F^2+\frac{\mu }{2}\sum \limits_{i=1}^m{\left\Vert \left(C\odot W\right)e\right\Vert}_2^2 $$

$$ s.t.\left(C\odot W\right)e=e,W\ge 0 $$

where *C* is an indicator matrix. *C*(*i*, *j*) = 1 if *x*_*j*_ ∈ *N*(*x*_*i*_); else *C*(*i*, *j*) = 0; *C*(*i*, *i*) = 0. ‖∙‖_*F*_ is the Frobenius-norm. *e* = (1, 1, …, 1)^*T*^, and ⊙ is Hadamard product. *μ* is the tradeoff parameter. *W* is a *m* × *m* weight matrix, where the *i*th row indicates the data points’ reconstruction contributions to the data point *x*_*i*_.

To solve the objection function (1), we introduce the Lagrange function:

2 $$ L=\frac{1}{2}{\left\Vert X-\left(C\odot W\right)X\right\Vert}_F^2+\frac{\mu }{2}{\left\Vert \left(C\odot W\right)e\right\Vert}_2^2-{\lambda}^T\left(\left(C\odot W\right)e-e\right)- tr\left({\varPhi}^T\ W\right) $$

where Φ is Lagrange multiplier. Differentiating *L* with respect to *W*, we have:

$$ {\nabla}_WL=C\odot \left(\left(C\odot W\right)X{X}^T+\mu \left(C\odot W\right)e{e}^T-X{X}^T-\lambda {e}^T\right)-{\varPhi}^T $$

By Complementary slackness condition, we obtain:

$$ {\left(\left(C\odot W\right)X{X}^T+\mu \left(C\odot W\right)e{e}^T-X{X}^T-\lambda {e}^T\right)}_{ij}{W}_{ij}{C}_{ij}=0 $$

So *W*_*ij*_ can be written as:

3 $$ {W}_{ij}=\left\{\begin{array}{c}\frac{W_{ij}{\left(X{X}^T+\lambda {e}^T\right)}_{ij}}{{\left(\left(C\odot W\right)X{X}^T+\mu \left(C\odot W\right)e{e}^T\right)}_{ij}}\ {x}_j\in N\left({x}_i\right)\\ {}0\kern5.50em {x}_j\notin N\left({x}_i\right)\end{array}\right. $$

But there still exists *λ* in (3), and (2) has the equivalent form:

4 $$ \underset{\omega_i}{\mathit{\min}}{L}^i=\frac{1}{2}{\left\Vert {x}_i-{\sum}_{i_j:{x}_{i_j}\in N\left({x}_i\right)}{\omega}_{i,{i}_j}\ \right\Vert}^2+\frac{\mu }{2}\ {\left({\sum}_{i_j:{x}_{i_j}\in N\left({x}_i\right)}\left|{\omega}_{i,{i}_j}\right|\right)}^2=\frac{1}{2}{\omega_i}^T{G}^i{\omega}_i+\frac{\mu }{2}{\left\Vert {\omega}_i\right\Vert}_1^2 $$

$$ s.t.{e}^T{\omega}_i=1,{\omega}_i\ge 0 $$

where *G*^*i*^ is the Gramm Matrix whose entry is $\left({x}_i,{x}_{i_j}\right){\left({x}_i,{x}_{i_k}\right)}^T$. The Lagrange function of (4) is:

5 $$ {L}^i=\frac{1}{2}{\omega}_i^T{G}^i{\omega}_i+\frac{\mu }{2}{\left\Vert {\omega}_i\right\Vert}_1^2-{\lambda}_i\left({e}^T{\omega}_i-1\right)-{\eta}^T{\omega}_i $$

By Karush–Kuhn–Tucker (KKT) conditions, we obtain:

$$ \left\{\begin{array}{c}\kern3.75em {\nabla}_{\omega_i}{L}^i={G}^i{\omega}_i+\mu e{e}^T{\omega}_i-{\lambda}_ie-\eta =0\\ {}{\nabla}_{\lambda_i}{L}^i={e}^T{\omega}_i-1=0\ \\ {}\eta \ge 0,{\omega}_i\ge 0,{\eta}_j{\omega}_{i,{i}_j}=0\ \end{array}\right. $$

Then, it can be inferred that:

$$ {\omega}_i^T{\nabla}_{\omega_i}{L}^i={\omega}_i^T{G}^i{\omega}_i+\mu {\left({\omega}_i^Te\right)}^2-{\lambda}_i{\omega}_i^Te=0 $$

So:

$$ {\lambda}_i=\left({\omega}_i^T{G}^i{\omega}_i+\mu {\left({e}^T{\omega}_i\right)}^2\right)/{e}^T{\omega}_i $$

The reconstruction error $\frac{1}{2}{\omega}_i^T{G}^i{\omega}_i\approx 0$. If *ω*_*i*_ is the optimal solution for (5), *e*^*T*^*ω*_*i*_ − 1 = 0. So *λ*_*i*_ ≈ *μ*. Let *λ* = *μe*. Then we obtain:

6 $$ {W}_{ij}=\left\{\begin{array}{c}\frac{W_{ij}{\left(X{X}^T+\mu e{e}^T\right)}_{ij}}{{\left(\left(C\odot W\right)X{X}^T+\mu \left(C\odot W\right)e{e}^T\right)}_{ij}}\kern0.50em {x}_j\in N\left({x}_i\right)\\ {}\kern4.5em \ 0\kern1em {x}_j\notin N\left({x}_i\right)\end{array}\right. $$

Weight matrix *W* is updated according to Eq. (6) until convergence.

### Sequence similarity and interaction profile similarity

In this section, we introduce mathematical notations for lncRNA (and miRNA) interaction profile, lncRNA (and miRNA) sequence similarity and lncRNA (and miRNA) interaction profile similarity. Given lncRNAs *L*_1_, …, *L*_*i*_, …, *L*_*l*_ and miRNAs *M*_1_, …, *M*_*j*_, …, *M*_*m*_, their pairwise interactions are represented by a *l* × *m* interaction matrix *Y*, where *Y*_*ij*_ = 1 if the lncRNA *L*_*i*_ interacts with the miRNA *M*_*j*_, otherwise *Y*_*ij*_ = 0. By using the interaction matrix *Y*, we define the interaction profiles for lncRNAs and miRNAs. The interaction profile of lncRNA *L*_*i*_ is a binary vector specifying the absence or presence of its interactions with every miRNA, and corresponds to the *i* th row of *Y*, namely *Y*(*i*, :). The interaction profile of a miRNA *M*_*j*_ is a binary vector encoding the absence or presence of its interactions with every lncRNA, and corresponds to the *j* th row of *Y*, namely *Y*(:, *j*).

LncRNA sequences and miRNA sequences provide important information for exploring their functions, and the *k*-mer [38] is a popular sequence-derived feature, which describes repeated patterns of sequences. There exist four types of nucleotides i.e. A, C, G and T/U for both lncRNA sequences and miRNA sequences. For the *k*-mer feature, we count the frequencies of 4^*k*^ types of *k*-length contiguous subsequences along lncRNA (miRNA) sequences. More specifically, for a lncRNA (or miRNA) sequence *x*, the *k*-mer feature of the sequence is defined as ${f}_k(x)=\left({d}_1,{d}_2,\dots {d}_{4^k}\right)$, where *d*_*i*_ is the occurrence frequency of corresponding *k*-length contiguous subsequences. In this work, we set *k* = 5, and we present lncRNAs and miRNAs with their corresponding *k*-mer vectors. Then, we calculate sequence similarities for *l* lncRNAs, denoted as a *l* × *l* matrix *S*_*LSF*_, by using the linear neighborhood similarity measure (LNS). Similarly, we utilize LNS to calculate sequence similarities for *m* miRNAs, denoted as a *m* × *m* matrix *S*_*MSF*_.

Related studies [39–41] adopt biological molecules’ interaction profiles in prediction models and achieve high-accuracy performance. These studies reveal the importance of interaction profiles in predicting unknown associations. Based on the interaction matrix *Y*, lncRNAs *L*_1_, …, *L*_*i*_, …, *L*_*l*_ are represented by interaction profiles *Y*(1, :), …, *Y*(*i*, :), …, *Y*(*l*, :), and miRNAs *M*_1_, …, *M*_*j*_, …, *M*_*m*_ are represented by interaction profiles *Y*(:, 1), …, *Y*(:, *j*), …, *Y*(:, *l*). Then, we can respectively calculate interaction profile similarities for *l* lncRNAs, denoted as a *l* × *l* matrix *S*_*LIP*_, using the linear neighborhood similarity measure; we calculate interaction profile similarities for *m* miRNAs, denoted as a *m* × *m* matrix *S*_*MIP*_.

### Sequence-derived linear neighborhood propagation method

**Fig. 1:**
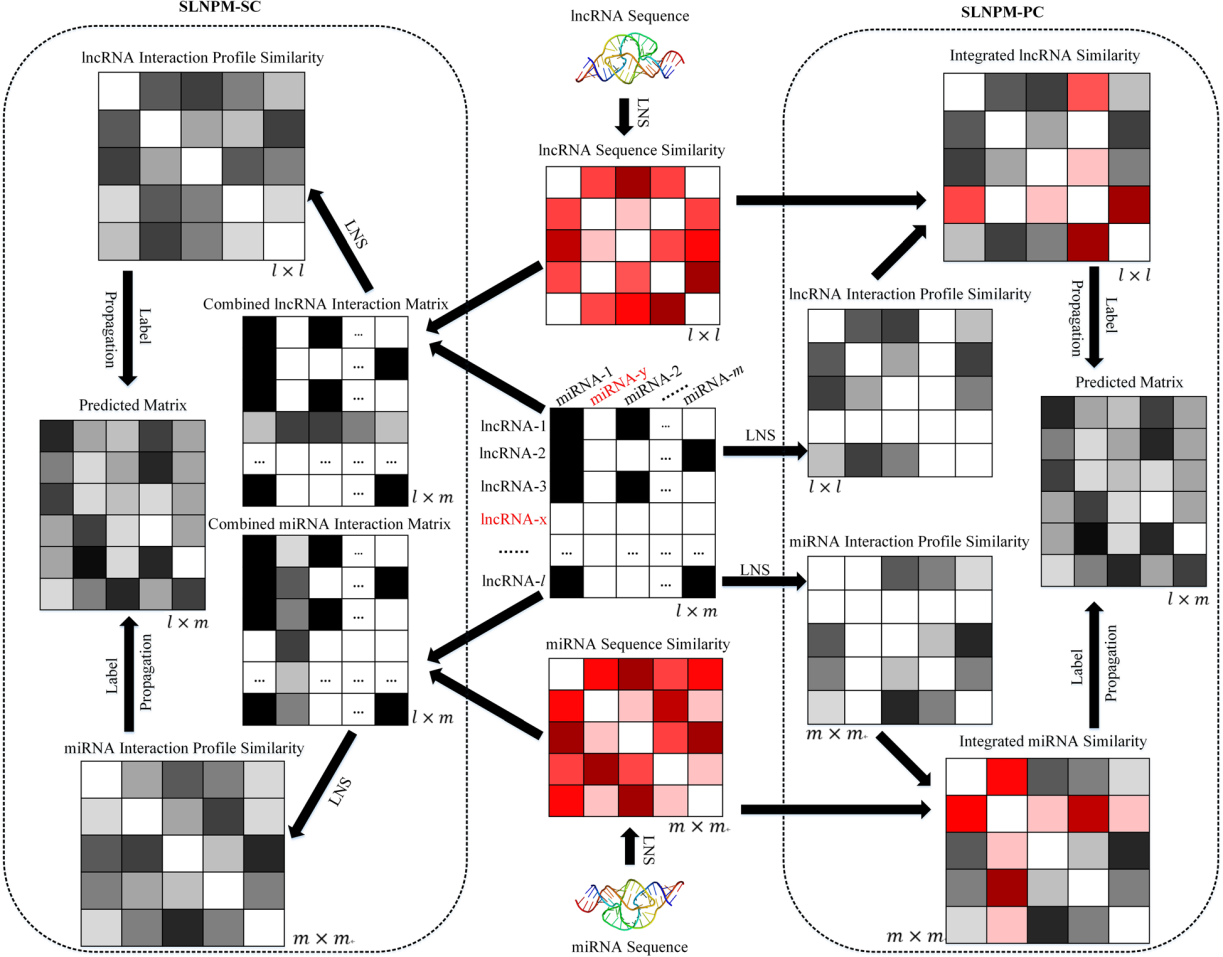
Workflow of the sequence-derived linear neighborhood propagation method. The figure explains two models: SLNPM-SC and SLNPM-PC. SLNPM-SC integrates sequence similarity and interaction profile similarity to obtain combined similarities, and then makes predictions based on the combined similarities; SLNPM-PC utilizes the sequence similarities to complement the interaction profiles, and then calculates the interaction profile similarity to make predictions

Since we have the sequence feature and interaction profiles for lncRNAs (miRNAs), we integrate diverse information of lncRNAs (or miRNAs) to develop prediction models. On the one hand, information integration can lead to improved performances. On the other hand, there exist lncRNAs (miRNAs) that have no known interaction with any miRNA (lncRNA), and the interaction profiles are unavailable for these lncRNAs (miRNAs). The information integration can deal with such lncRNAs (miRNAs). Here, we propose a sequence-derived linear neighborhood propagation method (SLNPM) and consider two strategies: similarity-based information combination (SC) and interaction profile-based information combination (PC) to integrate diverse features and meanwhile address above-mentioned problems. Thus, we present two editions of SLNPM: sequence-derived linear neighborhood propagation method based on similarity information combination (SLNPM-SC) and sequence-derived linear neighborhood propagation method based on interaction profile information combination (SLNPM-PC). The flowchart of two prediction models is shown in Fig. 1.

### Similarity-based information combination

In this section, we propose the similarity-based information combination strategy to build the sequence-derived linear neighborhood propagation model, abbreviated as SLNPM-SC.

For a lncRNA *L*_*i*_ (miRNA *M*_*j*_), which has no interaction with any miRNA (lncRNA), its interaction profile is an all-zero vector. We cannot calculate the interaction profile similarities for lncRNAs (miRNAs) without interactions. Therefore, entries in the *i* th (*j* th) row and *i* th (*j* th) column of the lncRNA (miRNA) interaction profile similarity matrix *S*_*LIP*_ (*S*_*MIP*_) are all zeros. The similarity-based information combination strategy is described below.

First, we calculate the sequence similarity *S*_*LSF*_ for all lncRNAs, and calculate the interaction profile similarity *S*_*LIP*_ for lncRNAs with interaction information. Then, we calculate the integrated similarity *S*_*LIS*_ for lncRNAs by:

7 $$ {S}_{LIS}\left(i,:\right)=\left\{\begin{array}{cc}{S}_{LIP}\left(i,:\right)& if\ {L}_i\  has\  interactions\ \\ {}{S}_{LSF}\left(i,:\right)& otherwise\end{array}\ \right. $$

Similarly, we calculate the sequence similarity *S*_*MSF*_ for all miRNAs, and calculate the interaction profile similarity *S*_*MIP*_ for miRNAs with interaction information. Then, we calculate the integrated similarity *S*_*MIS*_ for miRNAs by:

8 $$ {S}_{MIS}\left(j,:\right)=\left\{\begin{array}{cc}{S}_{MIP}\left(j,:\right)& if\ {M}_j\  has\  interactions\ \\ {}{S}_{MSF}\left(j,:\right)& otherwise\end{array}\right. $$

Then, we construct a directed graph based on the integrated lncRNA similarity matrix *S*_*LIS*_, and construct another directed graph based on the integrated miRNA similarity matrix *S*_*MIS*_. Considering miRNA *M*_*j*_, the *j* th column of interaction matrix *Y* is regarded as the initial labels of all nodes (lncRNAs) in the integrated lncRNA similarity-based graph. The label information is iteratively propagated in the graph until convergence, and the details about label propagation can refer to [42]. The prediction matrix *P*^*l*^ with size *l* × *m* is obtained. Similarly, considering lncRNA *L*_*i*_, the *i*th row of interaction matrix *Y* is regarded as the initial labels of all nodes (miRNAs) in the integrated miRNA similarity-based graph, and the *l* × *m* prediction matrix *P*^*m*^. Finally, the prediction result of SLNPM-SC model is produced by:

9 $$ {P}_{\mathrm{SLNPM}-\mathrm{SC}}=\beta {P}^l+\left(1-\beta \right){P}^m $$

where 0 ≤ *β* ≤ 1 is the weighted coefficient.

### Interaction profile-based information combination

In this section, we propose the interaction profile-based information combination strategy to build a sequence-derived linear neighborhood propagation model, abbreviated as SLNPM-PC.

The interaction profiles of lncRNAs (miRNAs) without any interaction information are unavailable, and corresponding rows (columns) in the interaction matrix are all zeros. The interaction profile-based information integration strategy is described below.

For miRNA *L*_*i*_, which does not have any interaction, its interaction profile is complemented by the sequence information,

10 $$ Y\left(i,:\right)=\frac{1}{Q_i}{\sum}_{i_k\epsilon N\left({L}_i\right)}{S}_{LSF}\left(i,{i}_k\right)Y\left({i}_k,:\right) $$

where *N*(*L*_*i*_) is the set of *k* most similar lncRNAs to the lncRNA *L*_*i*_ based on lncRNA sequence similarity *S*_*LSF*_, and each of similar lncRNAs has at least one association with miRNAs. *Q*_*i*_ is the sum of similarity between the lncRNA *L*_*i*_ and *k* most similar lncRNAs, ${Q}_i={\sum}_{i_k\epsilon N\left({L}_i\right)}{S}_{LSF}\left(i,{i}_k\right)$.

Similarly, for miRNA *M*_*j*_, which does not have any interaction, its interaction profile is complemented by the sequence information,

11 $$ Y\left(:,j\right)=\frac{1}{Q_j}{\sum}_{j_k\epsilon N\left({M}_j\right)}{S}_{MSF}\left(j,{j}_k\right)Y\left(:,{j}_k\right)\kern0.5em $$

where *N*(*M*_*i*_) is the set of *k* most similar miRNAs for the miRNA *M*_*j*_ based on miRNA sequence similarity *S*_*MSF*_, and each of similar miRNAs has at least one association with lncRNAs. *Q*_*j*_ is the sum of similarity between the miRNA *M*_*j*_ and *k* most similar miRNAs, ${Q}_j={\sum}_{j_k\epsilon N\left({M}_j\right)}{S}_{MSF}\left(j,{j}_k\right)$.

After complementing interaction profiles by using lncRNA (miRNA) sequence similarities, we can calculate interaction similarity matrices for lncRNA and miRNA respectively. Then, we construct prediction models based on lncRNA-lncRNA similarity graph and miRNA-miRNA similarity graph by using label propagation, and the prediction models produce the prediction matrices *P*^*m*^ and *P*^*l*^. The final prediction matrix *P*_SLNPM − PC_ is produced by a weighted average of two prediction matrices,

12 $$ {P}_{\mathrm{SLNPM}-\mathrm{PC}}=\beta {P}^l+\left(1-\beta \right){P}^m $$

where 0 ≤ *β* ≤ 1 is the weighted coefficient.

## Results and discussion

### Evaluation metrics

Here, we adopt 5-fold cross-validation (5-CV) to evaluate prediction models. Specifically, we randomly split known lncRNA-miRNA interactions into five subsets. In each fold, we keep one subset as the testing set, and use others as the training set. All the prediction models are built on the interactions in the training set, and then make predictions for other lncRNA-miRNA pairs. Then, the predictions and real labels (interactions or not) for these pairs are used to calculate evaluation metrics: the area under receiver-operating characteristic curve (AUC), the area under precision-recall curve (AUPR), sensitivity (SEN), specificity (SPEC), precision (PREC), accuracy (ACC) and F-measure (F).

The area under the precision-recall curve (AUPR) and the area under the ROC curve (AUC) are adopted as the evaluation metrics. AUPR and AUC evaluate the performances of prediction models regardless of any threshold. We also adopt binary classification metrics to measure the models, i.e. recall (REC), specificity (SP), precision (PR), accuracy (ACC) and F1-measure (F1). In the experiments, we run 20 runs of 5-CV for each model and adopt averages.

### Parameter settings

In this study, both SLNPM-SC and SLNPM-PC have two major components: the linear neighborhood similarity calculation and similarity-based label propagation. The linear neighborhood similarity has the parameter: neighbor number *K*, and the label propagation has the parameter: absorbing probability *α*. *β* is a tradeoff parameter in the final prediction phase. Here, we consider different combinations of following values: {10%, 20%, 30%, …, 90%} of number of data points for *K*, {0.1, 0.2, 0.3, …, 0.9} for *α* and {0, 0.05, 0.1, …, 0.95, 1} for *β* to build SLNPM-SC model and SLNPM-PC model, and then evaluate the influence of parameters. All the experiments are conducted with 5-fold cross-validation on SLNPM-S dataset. The result shows that SLNPM-SC model achieves the best AUPR score of 0.6033 when *K* = 80%, *α* = 0.4 and *β* = 0.25 and SLNPM-PC model produces the best AUPR score of 0.5996 when *K* = 90%, *α* = 0.4 and *β* = 0.25.

**Fig. 2:**
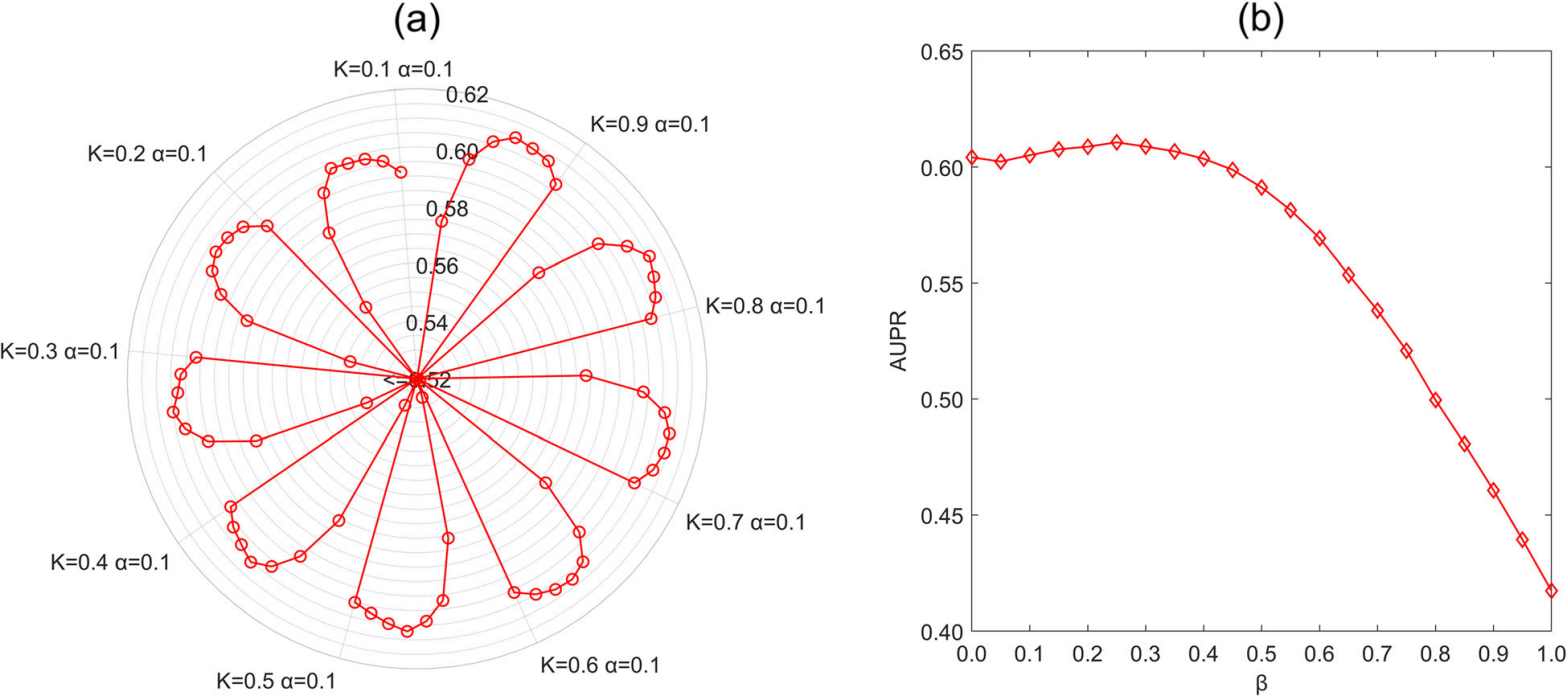
The influence of parameters on AUPR scores of SLNPM-SC model. **a** the influences of *K* and α when fixing β. **b** the influences of β when fixing *K* and α

For simplicity, we use the parameter setting in the SLNPM-SC model for analysis. Firstly, we set *β* = 0.25 and then evaluate the influence of *K* and *α* on the performances of SLNPM-SC model. The AUPR scores of SLNPM-SC models with different combinations of *K* value and *α* value are visualized in Fig. 2 (a). This figure indicates that the parameter *α* has great impact on the performance of SLNPM-SC model. More specifically, when *α* becomes greater, the performances first increase and then decrease after a peak. Besides, better performance can also be obtained as the neighborhood ratio *K* keeps increasing. This result might be the consequence of more neighbors’ information being considered to calculate similarity. Then, we fix *K* = 0.8 and *α* = 0.4 and evaluate the influence of parameter *β* in the prediction model. Note that *β* is a tradeoff parameter between lncRNA-based prediction and miRNA-based prediction. The parameter *β* = 1 means that SLNPM-SC only utilizes the lncRNA-lncRNA similarity information in lncRNA-miRNA interaction prediction. Vice versa, SLNPM-SC only uses the miRNA-miRNA similarity information when *β* = 0. All the results are summarized and shown in Fig. 2 (b) and denote that the prediction model produces the best result when *β* = 0.25. This result demonstrates the SLNPM-SC model depends more on the miRNA information-based component than the lncRNA information-based component (0.75 VS. 0.25).

Therefore, we adopt *K* = 80%, *α* = 0.4 and *β* = 0.25 for SLNPM-SC model and *K* = 90%, *α* = 0.4 and *β* = 0.25 for the SLNPM-PC model in the following sections.

### Results of SLNPM-SC and SLNPM-PC

SLNPM-SC integrates sequence similarity and interaction profile similarity to obtain combined similarities, and then makes predictions based on the combined similarities; SLNPM-PC utilizes the sequence similarities to complement the interaction profiles and then calculates the interaction profile similarity to make predictions.

To demonstrate the superiority of the SLNPM-SC and SLNPM-PC, we build several similar models by using individual features or other similarity measures. First, we respectively build sequence-derived linear neighbor propagation (SLNPM) models based on either interaction profile similarities or sequence similarities. Since existing work [43] ever used the expression profiles of lncRNAs and miRNAs in predicting lncRNA-miRNA interactions, we calculate the expression profile similarity by using linear neighborhood similarity measure (LNS) and build the SLNPM model. We also calculate the sequence similarity by using the Smith-Waterman algorithm (SW) [44] and build the SLNPM model. The performances of the above models are evaluated on SLNPM-S dataset by using 5-CV, and results are shown in Table 2. Clearly, SLNPM-SC and SLNPM-PC produce better results than other SLNPM models, indicating the effectiveness of two information combination strategies. The SLNPM model produced by LNS has better performances than the SLNPM model produced by SW, demonstrating the LNS can better measure lncRNA-lncRNA similarity and miRNA-miRNA similarity than SW. Moreover, the SLNPM models which utilize interaction profile similarities outperform other SLNPM models based on individual feature similarities, revealing the importance of interaction profiles.

**Table 2:** Performances of SLNPM models based on different information sources

Information Source	Similarity Computing	AUPR	AUC	REC	SP	PR	ACC	F1
Expression Profiles	LNS	0.0305	0.6981	0.0415	0.9974	0.0763	0.9935	0.0518
Sequences	SW	0.1358	0.8245	0.2515	0.9956	0.1989	0.9925	0.2191
	LNS	0.1856	0.8596	0.2883	0.9962	0.2436	0.9932	0.2621
Interaction Profiles	LNS	0.5981	0.8756	0.5993	0.9990	0.7180	0.9973	0.6500
SLNPM-SC		0.6033	0.9115	0.6043	0.9989	0.7028	0.9972	0.6469
SLNPM-PC		0.5996	0.9006	0.6092	0.9989	0.7087	0.9973	0.6522

Previous studies [26, 29] and our experimental results demonstrate that interaction profiles are critical for predicting lncRNA-miRNA associations. However, interaction profiles of some lncRNAs (miRNAs) are unavailable. Therefore, the models which mainly rely on interaction profiles cannot make predictions for such lncRNAs (miRNAs), and thus we solve this problem with the proposed information combination strategies which utilize the biological feature: lncRNA (miRNA) sequences. Besides, we notice that expression profiles can also describe lncRNAs (miRNAs), and relevant study [28] shows expression profiles play a crucial role in lncRNA-miRNA interactions. To compare the effectiveness of different information sources used in the combination strategy, we respectively utilize sequences and expression profiles to build SLNPM-SC and SLNPM-PC. The performances of these models are evaluated by 5-CV and detailed results are displayed in Table 3. Specifically, we calculate the lncRNA expression profile similarity and miRNA expression profile similarity by using linear neighborhood similarity measure, and build SLNPM-SC (M2) model and SLNPM-PC model (M4), our original SLNPM-SC model(M1) and SLNPM-PC model(M3) based on sequence similarity are denoted by M1 and M3 respectively. Clearly, the SLNPM models based on the sequence similarity can lead to much better performances than the SLNPM models based on expression profile similarity.

**Table 3:** Performances of SLNPM models based on different similarities combinations

Models	Model and Information Source	AUPR	AUC	REC	SP	PR	ACC	F1
M1	SLNPM-SC (combining sequence similarity)	0.6033	0.9115	0.6043	0.9989	0.7028	0.9972	0.6469
M2	SLNPM-SC (combining expression profile similarity)	0.3962	0.9000	0.5669	0.9973	0.4734	0.9955	0.5135
M3	SLNPM-PC (complementing IP with sequence similarity)	0.5996	0.9006	0.6092	0.9989	0.7087	0.9973	0.6522
M4	SLNPM-PC (complementing IP with expression profile similarity)	0.5236	0.8980	0.5787	0.9983	0.5929	0.9966	0.5843

*IP* interaction profile

Since we implement 20 runs of 5-CV for each model, we can obtain 20 AUPR scores and 20 AUC scores of each model. Further, we test the statistical difference between SLNPM-SC models (M1 and M2) and SLNPM-PC models (M3 and M4) by using the paired t-test. For the SLNPM-SC models, the *P*-values are 7.97E-27 (M2 VS. M1) and 1.07E-10 (M2 VS. M1) respectively in terms of the AUPR scores and AUC scores. For the SLNPM-PC models, considering the AUPR scores and AUC scores, the *P*-values are 1.24E-22 (M3 VS. M4) and 1.63E-04 (M3 VS. M4), respectively. The experimental results show that two editions of sequence-derived linear neighborhood propagation method (M1 and M3) can statistically outperform the SLNPM models based on expression information (M2 and M4) in terms of AUPR and AUC (*P*-value< 0.05).

### Comparison with state-of-the-art methods

To the best of our knowledge, there are only a few machine-learning based methods for lncRNA-miRNA interaction prediction. Here, we adopt EPLMI [26] and INLMI [28] as benchmark methods. EPLMI is a two-way diffusion model which uses the known lncRNA-miRNA interaction-based bipartite graph and expression profiles to predict lncRNA-miRNA interaction. We implement EPLMI using its publicly available source code. INLMI [28] integrates the expression similarity network and the sequence similarity network to predict lncRNA–miRNA interactions, and we implement this model according to descriptions in [28]. Since predicting lncRNA-miRNA interactions can be considered as a link prediction task, we adopt several network link inference methods as baseline methods, i.e. the collaborative filtering method (CF) [45] and the resource allocation algorithm (RA) [46]. The collaborative filtering method takes known lncRNA-miRNA interactions as a bipartite graph and exploits external information, such as expression profiles to calculate the lncRNA-lncRNA similarity and miRNA-miRNA similarity. Then, CF method finds neighbors for each lncRNA and each miRNA, and then predicts unknown interactions by utilizing a weighted average of its neighbors’ interacting miRNAs/lncRNAs, then combines the lncRNAs’ neighbors-based prediction and the miRNAs’ neighbor-based prediction with a tradeoff parameter. The resource allocation algorithm also formulates lncRNAs/miRNAs as nodes and lncRNA-miRNA interactions as links in a bipartite graph. Interaction information is iteratively propagated from miRNAs to their linked lncRNAs, and then the information is allocated from lncRNAs back to miRNAs. After finite iteration, final resources for miRNAs are probabilities that the lncRNA interacts with these miRNAs. EPLMI and RA have no parameter. INLMI has a parameter that represents the dimension of latent variable in the non-negative matrix factorization. CF has a trade-off parameter for the lncRNAs’ neighbor-based prediction and the miRNAs’ neighbor-based prediction. We tuned the parameters of INLMI and CF, and adopted the values that produce the best results.

All models are evaluated on SLNPM-S dataset by using 5-CV. As shown in Table 4, SLNPM-SC model achieves AUPR score of 0.6033 and AUC score of 0.9115, and SLNPM-PC model produces AUPR score of 0.5996 and AUC score of 0.9006. The performances of the proposed models are far better than EPLMI (AUPR score of 0.0706 and AUC score of 0.8494), INLMI (AUPR score of 0.0723 and AUC score of 0.8477), RA (AUPR score of 0.5078 and AUC score of 0.8637) and CF (AUPR score of 0.2363 and AUC score of 0.8610). There are several reasons why SLNPM-SC and SLNPM-PC have excellent prediction performances. On one hand, the linear neighborhood similarity measure effectively calculates the lncRNA-lncRNA similarities and miRNA-miRNA similarities. On the other hand, the integrated similarities and complemented interaction profile make use of diverse information.

**Table 4:** Performances of different models on SLNPM-S dataset

Methods	AUPR	AUC	REC	SP	PR	ACC	F1
EPLMI	0.0706	0.8494	0.1373	0.9962	0.0883	0.9939	0.1055
INLMI	0.0723	0.8477	0.1531	0.9956	0.0867	0.9935	0.1086
RA	0.5078	0.8637	0.5129	0.9987	0.6299	0.9967	0.5631
CF	0.2363	0.8610	0.4599	0.9956	0.3089	0.9934	0.3684
SLNPM-SC	0.6033	0.9115	0.6043	0.9989	0.7028	0.9972	0.6469
SLNPM-PC	0.5996	0.9006	0.6092	0.9989	0.7087	0.9973	0.6522

**Fig. 3:**
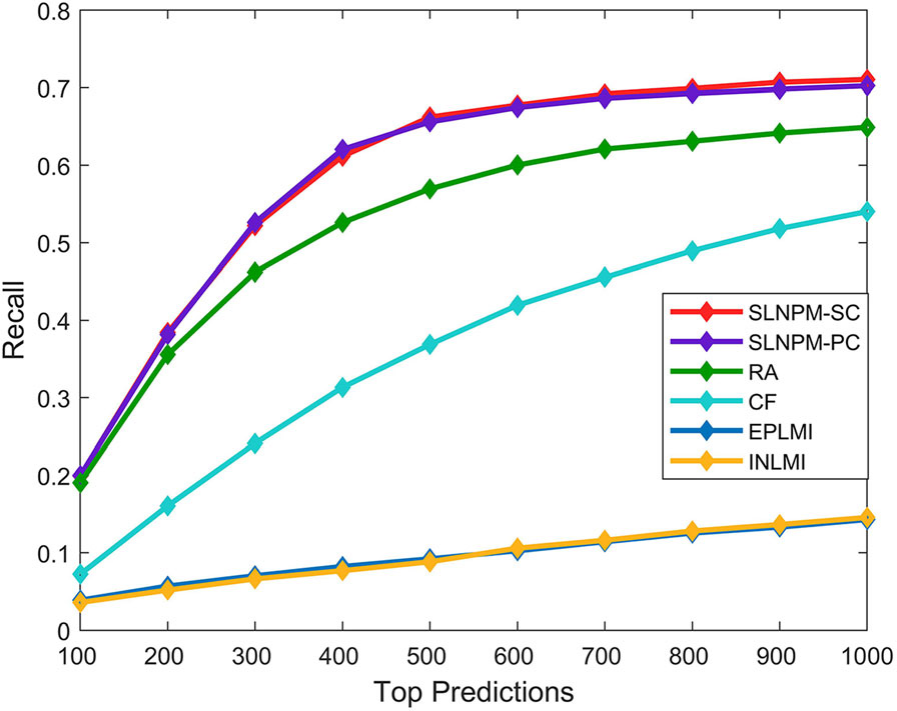
Recall of different methods in top-ranked predictions. The X-axis denotes the top predictions from the top 100 to the top 1000, and the Y-axis denotes the recall produced by SLNPM-SC and SLNPM-PC

In the computational predictions, the top-ranked predictions are very important and reflect the performances of models. Here, we check up on the top-ranked predictions ranging from top 100 to top 1000, and figure out how many real interactions can be predicted. As shown in Fig. 3, SLNPM-SC model and SLNPM-PC model perform better than the other three methods when checking up on top-ranked predictions. In the top 100 predictions, EPLMI, INLMI, RA, CF, SLNPM-SC and SLNPM-PC find out 18, 19, 87, 33, 91 and 91 real interactions respectively. Importantly, SLNPM-SC model and SLNPM-PC model can respectively predict 71 and 70% of interactions when only verifying top 1000 predictions.

### Case studies

In this section, we conduct the experiments on SLNPM-L dataset to demonstrate the practical capability of SLNPM-SC and SLNPM-PC for the lncRNA-miRNA interaction prediction.

**Fig. 4:**
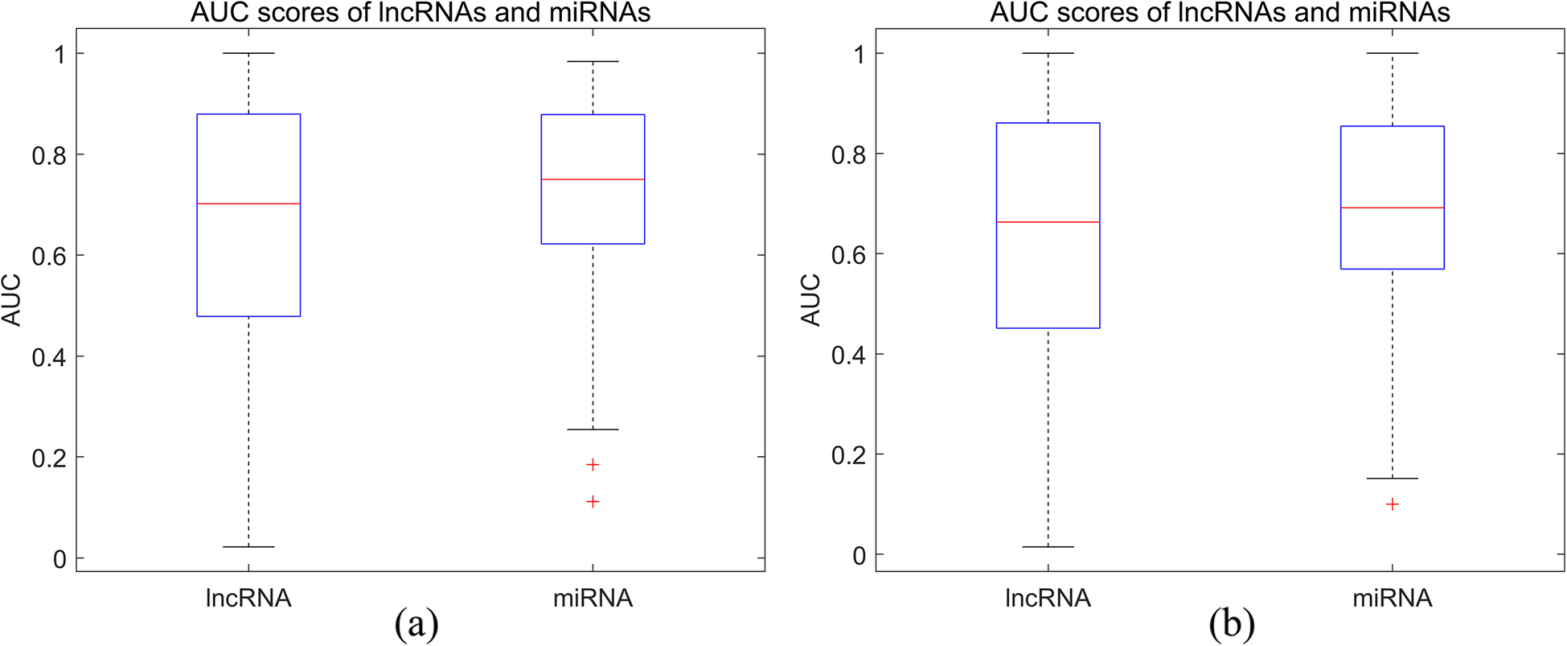
Boxplot of AUC scores for lncRNAs and miRNAs. **a** shows the boxplot of AUC scores of SLNPM-SC in predicting lncRNA-interacting miRNAs and miRNA-interacting lncRNAs. **b** shows the boxplot of the AUC scores of SLNPM-PC model in predicting lncRNA-interacting miRNAs and miRNA-interacting lncRNAs

First, we analyze the performances of SLNPM-SC and SLNPM-PC for predicting lncRNAs (miRNAs) interacted with a specific miRNA (lncRNA). In the experiment, we remove the interactions of a specific lncRNA or the interactions of a specific miRNA in our dataset, and build the SLNPM-SC model and SLNPM-PC model to predict the removed interactions. For every lncRNA or miRNA, we adopt the prediction scores and real labels (interaction or non-interaction) to calculate the AUC scores. We conduct the statistical analysis on the results for lncRNAs and miRNAs, and draw the boxplot. As shown in Fig. 4, the medians of lncRNAs and miRNAs are all larger than 0.65, indicating SLNPM-SC model and SLNPM-PC model can produce satisfying results in predicting lncRNA-interacting miRNAs and miRNA-interacting lncRNAs.

Further, we build the SLNPM-SC model and SLNPM-PC model based on SLNPM-L dataset to predict novel lncRNA-miRNA interactions, which are not included in the SLNPM-L dataset. Since the SLNPM-L dataset is compiled from lncRNASNP [17], the predictions are validated by other databases and publicly available literature. We take the lncRNA “MALAT1” and the miRNA “hsa-miR-17-5p” as examples, and respectively build prediction models (SLNPM-SC and SLNPM-PC) to predict miRNAs interacting with “MALAT1” and lncRNAs interacting with “hsa-miR-17-5p”. The lncRNA MALAT1(metastasis-associated lung adenocarcinoma transcript 1), a bona fide long noncoding RNA, is reported to be closely related with lung cancer and is one of the first discovered cancer-associated lncRNAs [47, 48]. The miRNA has-miR-17-5p, also known as miR-17, is identified as a member of solid cancer miRNA signature [49], and also acts as both an oncogene and a tumor suppressor in different cellular contexts [50, 51].

The top 10 predictions for the lncRNA “MALAT1” and the miRNA “hsa-miR-17-5p” are shown in Table 5. Both SLNPM-SC and SLNPM-PC correctly predict that hsa-miR-1 can interact with the lncRNA “MALAT1”. The study [60] reported that MALAT1 was identified as the target of miRNA hsa-miR-1, and MALAT1 could directly bind with hsa-miR-1, and level of miRNA hsa-miR-1 was negatively associated with that of MALAT1 in breast cancer tissues. In general, SLNPM-SC successfully identifies 5 miRNAs interacting with the lncRNA “MALAT1” and 4 lncRNAs interacting with the miRNA “hsa-miR-17-5p”; SLNPM-SC identifies 8 miRNAs interacting with the lncRNA “MALAT1” and 4 lncRNAs interacting with the miRNA “hsa-miR-17-5p”. Therefore, both SLNPM-SC and SLNPM-PC can predict novel lncRNA-miRNA interactions with high accuracy.

**Table 5:** Top 10 prediction of LNPM-SC and SLNPM-PC for lncRNA “MALAT1” and miRNA “hsa-miR-17-5p”

	SLNPM-SC				SLNPM-PC			
	MALAT1		hsa-miR-17-5p		MALAT1		hsa-miR-17-5p	
NO	miRNAs	Confirmed?	lncRNAs	Confirmed?	miRNAs	Confirmed?	lncRNAs	Confirmed?
1	hsa-miR-1	YES [52]	lnc-SNRPN-8	N.A.	hsa-miR-1	YES [52]	lnc-SNRPN-8	N.A.
2	hsa-miR-101-3p	YES [53]	KCNQ1OT1	N.A.	hsa-miR-101-3p	YES [53]	KCNQ1OT1	N.A.
3	hsa-miR-206	YES [54]	XIST	YES [55]	hsa-miR-142-3p	YES [56]	XIST	YES [55]
4	hsa-miR-210-3p	N.A.	lnc-COL9A2–1	N.A.	hsa-miR-206	YES [54]	lnc-COL9A2–1	N.A.
5	hsa-miR-216a-5p	YES [57]	lnc-ALYREF-1	N.A.	hsa-miR-210-3p	N.A.	lnc-ALYREF-1	N.A.
6	hsa-miR-329-3p	N.A.	COX10-AS1	YES [55]	hsa-miR-216a-5p	YES [57]	COX10-AS1	YES [55]
7	hsa-miR-335-5p	N.A.	lnc-NFAT5–2	N.A.	hsa-miR-335-5p	N.A.	lnc-NFAT5–2	N.A.
8	hsa-miR-3529-5p	N.A.	lnc-ACER2–1	YES [58]	hsa-miR-376b-3p	YES [52]	lnc-ACER2–1	YES [58]
9	hsa-miR-362-3p	N.A.	lnc-LUZP1–1	YES [55]	hsa-miR-455-5p	YES [59]	lnc-LUZP1–1	YES [55]
10	hsa-miR-376b-3p	YES [52]	lnc-NMRK1–1	N.A.	hsa-miR-876-5p	YES [52]	lnc-NMRK1–1	N.A.

*N.A* not available.

## Conclusions

LncRNA-miRNA interactions are critical to many biological events, and exploring these interactions contributes to understanding lncRNA’s functions. In this work, we propose a computational method named the sequence-derived linear neighborhood propagation method (SLNPM). SLNPM makes the best use of lncRNA sequences, miRNA sequences and known lncRNA-miRNA interactions to predict novel lncRNA-miRNA interactions. To deal with the miRNAs (or lncRNAs) without interaction information, we introduce two information combination strategies: similarity-based information combination and interaction profile-based information combination, and develop two editions of SLNPM: SLNPM-SC and SLNPM-PC. The proposed models are compared with benchmark methods and baseline methods. The experimental results show that the interaction profiles are very important for the high-accuracy performances of SLNPM-SC and SLNPM-PC, and the information combination strategies further improve performances. The prediction capabilities of proposed models are also tested by case studies, and predicted lncRNAs (miRNAs) for the given miRNA (lncRNAs) are confirmed by existing literature. In conclusion, SLNPM-SC and SLNPM-PC are promising for lncRNA-miRNA interaction prediction. However, SLNPM has several parameters, and it costs a large amount of time to determine optimal parameters. How to effectively tune parameters of SLNPM is our future consideration.

## Data Availability

Not applicable.

## Abbreviations

5-CV: 5-fold cross-validation
AUC: Area under ROC curve
AUPR: Area under the precision-recall curve
IP: Interaction profile
SLNPM-PC: Sequence-derived linear neighborhood propagation method based on interaction profile information combination
SLNPM-SC: Sequence-derived linear neighborhood propagation method based on similarity information combination
